# Study of noise and structural characteristics of twin-screw air compressor based on multi-physical field coupling

**DOI:** 10.1038/s41598-023-48362-4

**Published:** 2023-11-27

**Authors:** Yayin He, Congcong Xiao, Wei Zhang, Kai Wang

**Affiliations:** School of Mechanical Engineering, Shaanxi University of Technology, Hanzhong, 723000 Shaanxi China

**Keywords:** Energy science and technology, Engineering

## Abstract

For the problems of high noise and low efficiency of twin-screw air compressor, the mutual coupling effect of noise and structure for the twin-screw air compressor is studied by using acoustic-solid coupling technology. The simulation model of acoustic-structural coupling of twin-screw air compressor is established. Combining acoustic wave theory calculation with structural characteristics, the sound pressure level distribution and structural deformation law of different tangent planes of the shell at different frequencies under external excitation are studied. The results show that when the frequency is different, the more intensive the acceleration distribution on the shell surface is, the greater the sound pressure level is. At the same frequency, as the incident pressure wave increases, the deformation on the shell also increases, and the maximum shell deformation and the maximum sound pressure level both appear around 2000 Hz. As the frequency increases, the acceleration distribution on the surface of the shell becomes more denser and the sound pressure level becomes larger.

## Introduction

Twin-screw air compressors are widely used in the industrial field by virtue of their stable performance, high performance-price ratio, and large rotational speed. The working principle of twin-screw air compressors is that a pair of screw rotors compress air by changing the size of the volume between the teeth during the working process, and this process is very likely to cause noise. Due to the age of the twin-screw air compressor or installation errors, the bearings at the end of the twin-screw rotor shaft are misaligned, resulting in rotor instability or rotor grinding during work. This vibration condition can make the generated sound waves from the end of the screw rotor shaft to the shell cavity, and the repeated superposition of sound waves will cause mechanical noise and shell micro-deformation, which will reduce the operating efficiency and increasing energy consumption of the air compressor. In addition, there are many factors affecting the acoustic distribution of air compressors, such as shell structure, material, dimensional parameters and media, etc. In traditional air compressors, the shell material and oil medium are relatively fixed. As the air compressor studied in this project, its shell material and oil medium are relatively fixed. Therefore, it is very important to analyze the distribution law of noise size and the influence law of acoustic wave on structural deformation through simulation to optimize the structural design of air compressor, reduce the pneumatic noise, improve the operating efficiency, and reduce energy consumption. Therefore, it is very important to analyze the distribution law of noise size and the influence law of acoustic wave on structural deformation through simulation to optimize the structural design of air compressor, reduce the pneumatic noise, improve the operating efficiency, and reduce energy consumption.

At present, the theory and application of noise at home and abroad have become mature, and the research on noise problems of screw-type air compressors has gradually increased. In terms of air compressor noise research, Shen et al.^1^ achieved noise reduction of a semi-closed variable frequency twin-screw refrigeration air compressor using two methods, such as end-face attenuation channels and drain damping. Sangfors et al.^2^ proposed a computer simulation of pneumatic noise, that was generated by opening and closing the inlet and outlet of a twin-screw air compressor, moreover, they compared the calculated values with the experimental measurements. Mujic et al.^3^ analyzed the noise sources of twin-screw air compressors, identified the most important of them, and proposed methods to minimize their impact. Sun et al.^4^ compared twin-screw refrigeration air compressors with slide-valve control and variable-speed control, and investigated the relationship between their pressure fluctuations, noise characteristics and thermodynamic performance at the same volume flow rate, and the results showed that air compressors with variable-speed control have better overall performance than those with slide-valve control. Wang et al.^5^ proposed general steps and measurement procedures for noise and vibration evaluation in the primary stage of receiver testing when considering different loads. Yang et al.^6^ studied the effect of oil supply parameter variation on the vibration and performance of twin-screw air compressor bodies based on the mechanism of radiation noise generation from screw air compressor bodies, and at the same time, identified the main factors affecting the vibration and performance of the bodies. Wang et al.^7^ used CFX calculations to obtain the transient flow field of a double-suction balanced screw air compressor, thus obtained the relationship between the sound pressure level of the air compressor noise frequency and the exhaust frequency of the air compressor and its multiplication frequency.

A lot of work has also been done by domestic and foreign scholars on the performance analysis and control of air compressors. Hou et al.^8^ developed a thermodynamic model of the operating process of a water-injected twin-screw air compressor by considering both the effects of internal leakage and air–water heat transfer. Wang et al.^9^ measured the trend of gas pressure in the grooves of the compressor rotor under partial load to improve the stability of twin-screw air compressor operation. Li et al.^10^ studied the deformation and stress distribution law of the screw rotor of a twin-screw air compressor under temperature load. Ren HuanMei et al.^11^ made an optimized design for the profile of the twin-screw air compressor rotor, and at the same time, the performance of the pre- and post-optimized screw rotor was compared. Hou et al.^12^ built a twin-screw refrigeration compressor rotor shaft trajectory test system and conducted an experimental study on the shaft trajectory under different operating conditions as well as the inner volume ratio. Fan^13^ established an evaluation system of performance indicators combining fuzzy comprehensive evaluation method and hierarchical analysis method for the interrelationship between comprehensive performance and various parameters of twin-screw air compressor. Zhao et al.^14^ proposed three control methods applicable to the fixed speed control mode of the air compressor and applied the method to the integrated electric-gas energy flow calculation. Bo^15^ reviewed the status of research in the area of energy-efficient design of screw air compressors.

From the above, in recent years, it can be seen that the research on twin-screw air compressors at home and abroad has mainly focused on profile design, structural characteristics analysis, system control and noise analysis, while fewer studies have been conducted to combine the structure and noise of twin-screw air compressors. The transfer of sound pressure inside the compressor is determined by the structure of the compressor, part of the sound pressure is transmitted from the suction and exhaust ports of the compressor shell, part of the sound pressure is transmitted from the shell wall, while the other part of the sound pressure is reflected inside the cavity, and as the number of superimposed times increases, the energy of the sound wave is weakened. In this paper, based on the analysis of acoustic wave theory and structural characteristics of air compressor, a multi-physical field coupling method is used to analyze the acoustic-structural coupling of air compressor head, which is the core component of twin-screw air compressor. meanwhile, by simulating the excitation caused by bearing misalignment at both ends of the rotor caused by touch grinding or instability, the effect on noise variation and structure is analyzed.

## Acoustic-structural coupling calculation method

Although sound pressure is an important physical parameter for noise evaluation, however, the size of the sound pressure is directly related to the distance from the sound source and the environment in which it is measured, so the sound power level is usually used to measure the sound radiation capacity of a sound source. Specifically, the sound power level refers to the total energy radiated by the sound source to the entire space per unit of time, expressed in LW; sound pressure level is the most basic parameter reflecting the size and strength of the sound, expressed in Lp.

The sound power level can be defined as

1 $$ L_{W} = 10\lg \frac{W}{{W_{ref} }} $$

where $W$ is the sound power and $W_{ref}$ is the reference sound power. The relationship between the sound power level LW and the sound pressure level Lp is as follows.

2 $$ L_{W} = L_{p} + 20\lg r + K $$

where *r* is the distance of a point from the source (m), *K* is a constant. *K* value is related to the source propagation, when the source energy is spread around, then *K* is taken as 11 dB; when the source is placed in the ground easy to reflect, the energy can only be spread to the upper half of the air, then K is taken as 8 dB.

The acoustic-structural coupling model was established by using the "acoustic-solid interaction, frequency domain" multi-physical field coupling interface^16^. And the multi-physical field coupling interface contains two physical field interfaces, such as "solid mechanics" and "pressure acoustics, frequency domain", as well as the "acoustic-structural coupling boundary" coupling feature. First, the domain equations are established, and the sound waves in the air domain are modeled by the Helmholtz equation for sound pressure in the "pressure acoustics" feature^17^:

3 $$ \nabla \cdot \left( { - \frac{1}{{\rho_{c} }}\nabla p} \right) - \frac{{\omega^{2} }}{{\rho_{c} c^{2} }} = 0 $$

where the sound pressure is the harmonic quantity, $p = p_{0} e^{i\omega t}$,p is the pressure, $\rho_{c}$ is the density, $\omega$ is the angular frequency, $c$ is the speed of sound, and their units are N/m^2^, kg/m^3^, rad/s, m/s, respectively. The basic data of the acoustic domain are shown in Table 1.

**Table 1:** Basic parameters of acoustic domain.

Physical quantities	Values	Descriptions
*ρ*_*c*_ (kg/m^3^)	7850	Density
*c* (m/s)	1500	Sound velocity
*f* = *ω/*2π	*f*_*0*_	Frequency
*R* (m)	0.3	Model domain radius

In order to analyze the frequency response, the harmonic stresses and strains in the shell need to be calculated. The linear elastic material model is set up and the material is set to structural steel.

## Model building and solution setup

### Establishment of acoustic-structural coupling simulation model

**Figure 1:**
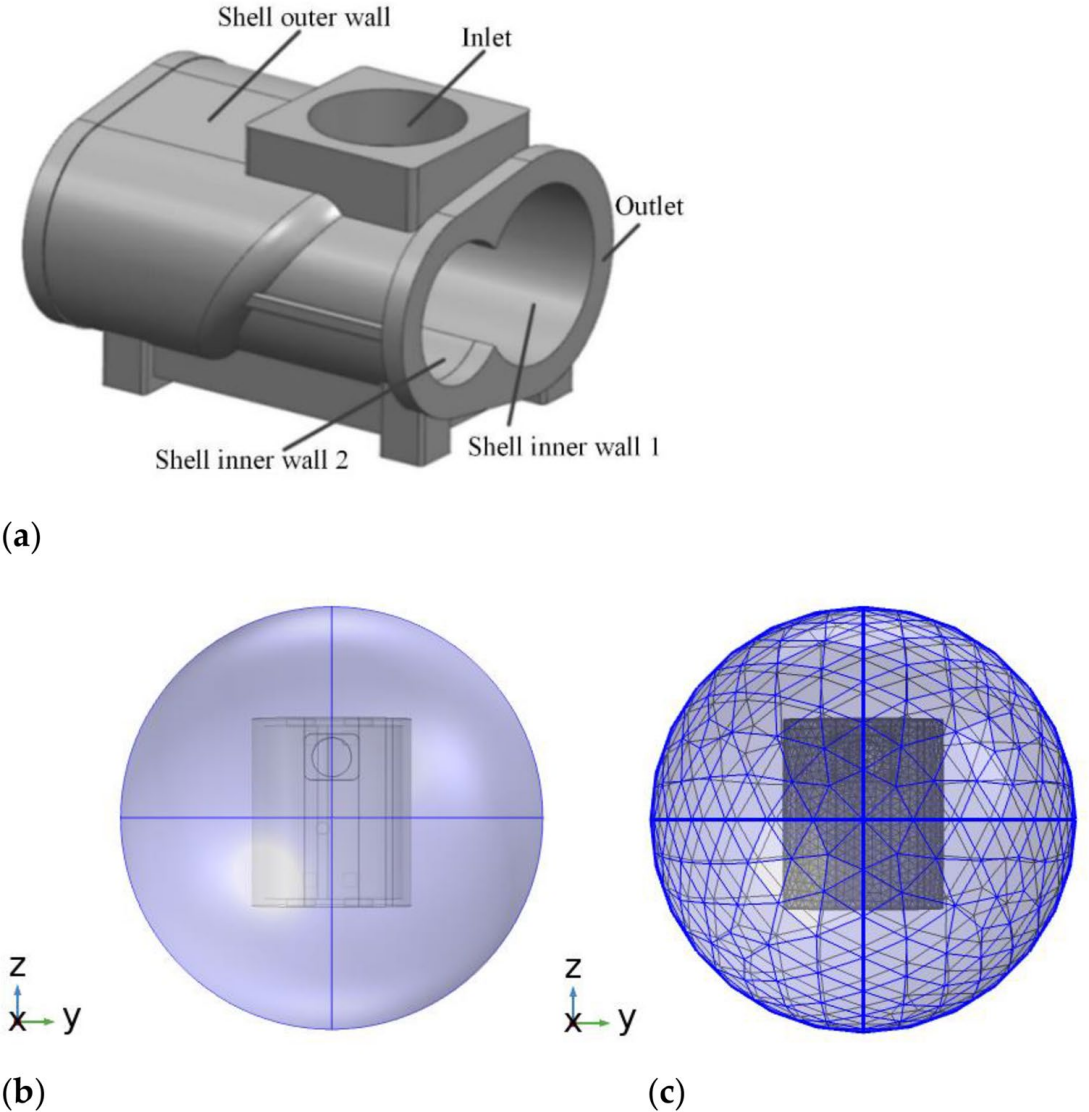
Sound structure coupling sound field model. (**a**) The 3Dmodel of air compressor shell, (**b**) Acoustic-structural coupling model and (**c**) Grid division.

The research object of this paper is a twin-screw air compressor with screw rotor model LGY03 of a factory, and the air compressor shell material is HT250. Due to the complex structure of the twin-screw air compressor shell, in order to facilitate the mesh division, save computational resources and shorten the computational time, the three-dimensional solid modeling process of the shell was appropriately simplified under the condition of meeting the actual requirements and not affecting the accuracy of the calculation. The shell design parameters are shown in Table 2, and the three-dimensional solid model of the shell is established using UG as shown in Fig. 1a. The established acoustic-structural coupling simulation model consists of two parts, respectively, the shell structure for the solid domain and the spherical shell for the fluid domain, as shown in Fig. 1b. After converting the model format and importing it into the software for meshing, the simulation mesh model of the coupled acoustic-structural model is shown in Fig. 1c.

**Table 2:** Twin-screw air compressor shell design parameters.

Shell parameters	Parameter value
Shell outer wall length/mm	268
Shell outer wall width/mm	258
Shell outer wall height/mm	208
Shell outer wall thickness/mm	12
Diameter of shell inner wall 1/mm	116
Diameter of shell inner wall 2/mm	92

The meshing is done using multi-physics field coupled simulation software, and different adapted meshes are required for different computational domains. For the fluid domain the maximum cell size is controlled by using the incident frequency as well as the wavelength size, and 6 grid cells are set for each wavelength, meanwhile the solid domain specifies the grid type as tetrahedral structure, and finally the whole mesh is obtained by two types of meshes.

### Boundary condition setting and solution setting

In Fig. 1, the incident plane wave is set as the incident acoustic wave on the outer spherical surface of the air domain, and after the acoustic wave interacts with the shell, the system allows the acoustic wave to leave in the form of a spherical wave. In the "Pressure Acoustics, Frequency Domain" interface, the incident scenario is implemented using a spherical wave radiation boundary, and this boundary condition can meet the calculation requirements in the case of air domain only around the shell. The conditions of the radiation boundary are set as shown in Table 3.

**Table 3:** Radiation boundary parameters.

Physical quantities	Values	Descriptions
$\hat{k}$	$\left( {\sin \theta \cos \theta ,\sin \theta \sin \varphi ,\cos \theta } \right)$	Incident wave direction vector
p_0_ (Pa)	1	Pressure amplitude

The incident wave direction is controlled by two angles: $0 < \theta < \pi$ and $0 < \varphi < 2\pi$.

Shell-air interface:

The coupling between the fluid domain and the solid is done by the “acoustic-structural boundary” coupling feature. The boundary load F on the shell is set to

4 $$ F = - n_{s} p $$

where* n*_*s*_ denotes the unit normal vector seen outward in the solid domain, while the normal acceleration of the fluid is set equal to the normal acceleration of the solid and can be expressed as

5 $$ a_{n} = - n_{a} \cdot \left( { - \frac{1}{{\rho_{0} }}\nabla p + q} \right) $$

As a comparison, the model can be simplified. The solid boundary in it is set as a hard sound field boundary, so that the sound does not affect the shell but affects the sound field distribution around the shell. In the model, this is achieved by setting fixed constraints on all solid boundaries, which become hard sound field boundaries when the boundary condition $a_{n} = 0$.

6 $$ n_{a} \cdot \left( { - \frac{1}{{\rho_{0} }}\nabla p + q} \right) = 0 $$

In Eqs. (5) and (6), $n_{a}$ is the unit normal vector seen outward from the acoustic domain, and the normal acceleration $a_{n}$ is equal to $\left( {n_{a} \cdot u} \right)\omega^{2}$, where u is the calculated harmonic displacement vector of the solid structure.

## Analysis of simulation results

The acoustic-structural coupling analysis of the air compressor shell was done by multi-physical field coupling technique. When the incident frequency is 1300 Hz, the air compressor shell characteristics, such as sound pressure level, deformation, and surface acceleration distribution are analyzed respectively. In order to obtain the distribution law of sound pressure level, deformation and surface acceleration on the shell surface after the incident acoustic wave acts with the shell, three coordinate axis directions are selected, and then three cross sections are selected in each direction.

### Sound pressure level, deformation, and surface acceleration distribution in each plane

**Figure 2:**
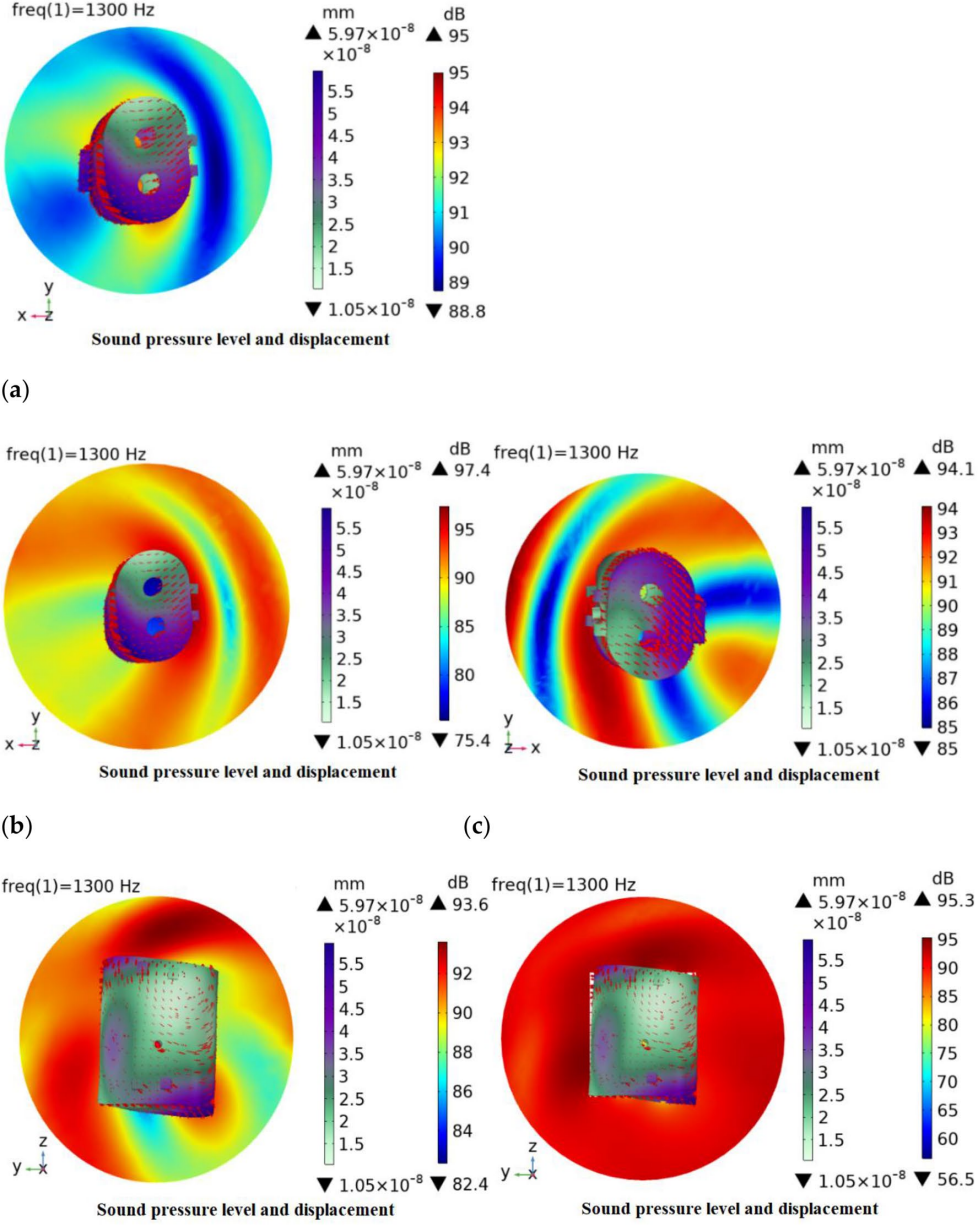

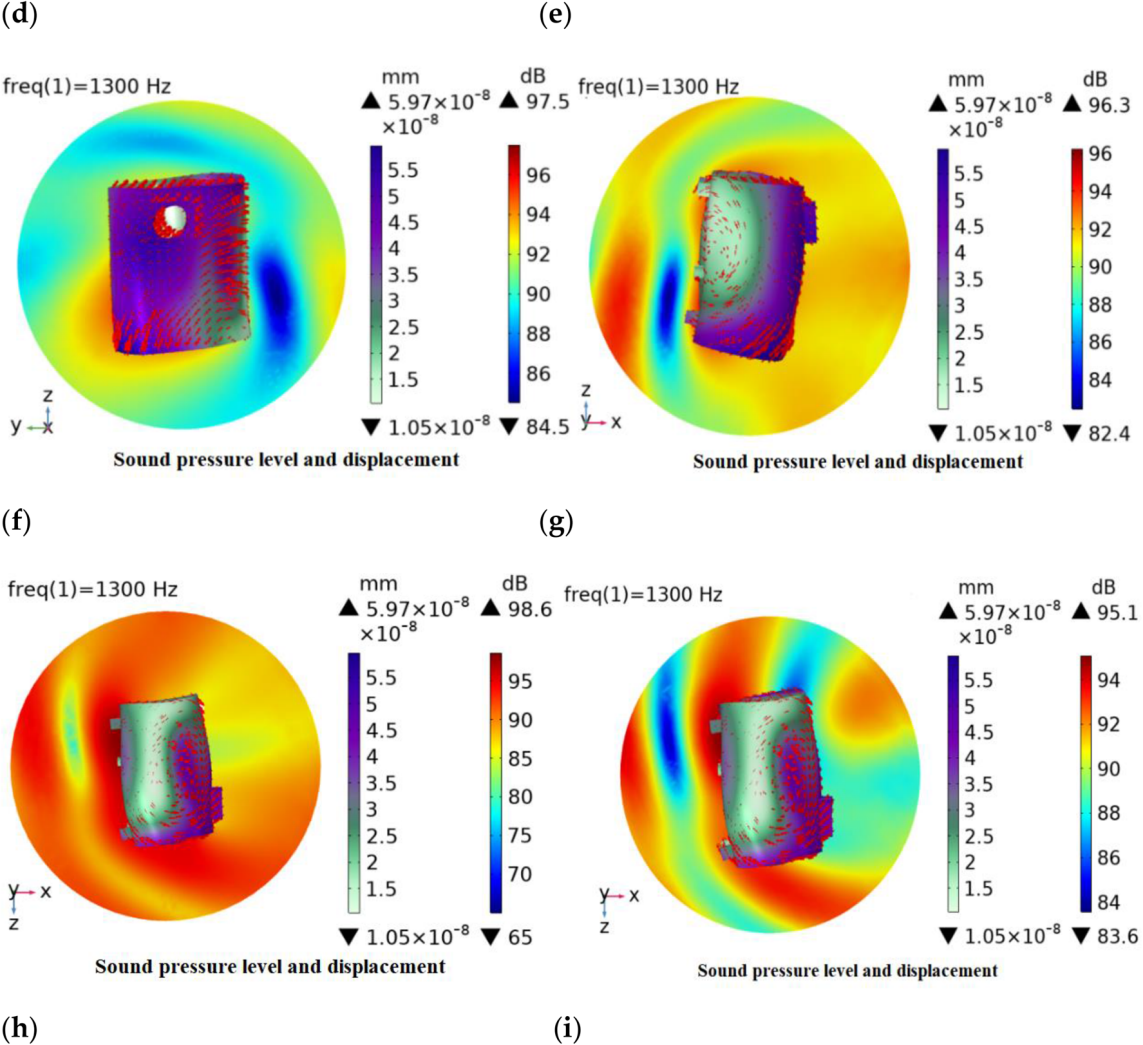
Sound structure coupling cloud diagram. (**a**) XY plane positive section, (**b**) XY plane intermediate section, (**c**) XY plane negative section, (**d**) YZ plane positive section, (**e**) YZ plane intermediate section, (**f**) YZ plane negative section, (**g**) ZX plane positive section, (**h**) ZX plane intermediate section and (**i**) ZX plane negative section.

The three planes XY, YZ and ZX were taken, and the three planes were taken as the reference, respectively, and the positive and negative tangents were taken in the respective parallel directions. The relationship between its sound pressure level, structural deformation and surface acceleration is analyzed, and the cloud diagram is shown in Fig. 2.

By analyzing the simulation results after the incident wave enters the acoustic domain, the results in Fig. 2 show that there are sound pressure level, deformation, and surface acceleration, where the red arrow represents the surface acceleration, and the length of the arrow represents the surface acceleration magnitude, which indicates the magnitude of the interaction between the acoustic wave and the cavity, and the intensity represents the frequency of the interaction between the surface acoustic wave and the cavity in that region. Combined with the three planes, as a whole, the excitation sound pressure level distribution of each plane is around its tangent to the surrounding dispersion, and the distribution of the sound pressure level in different tangents of the same plane is different.

Specifically, in the XY plane, the maximum sound pressure level is distributed in the middle section with a value of 97.4 dB, and the minimum sound pressure level also appears in the middle section with a value of 75.4 dB. In addition, the acceleration distribution near the front and rear axial end surfaces of the shell is relatively dense, indicating that there is more superposition of sound waves in this region, which has a greater impact on the sound pressure level distribution. In the YZ plane, the maximum sound pressure level is distributed in the negative section with a value of 97.4 dB, and the minimum sound pressure level appears in the middle section of the YZ plane with a value of 56.5 dB. The surface acceleration distribution in the inlet plane of the shell is relatively dense, indicating that the number of sound waves superimposed in this region has a greater influence on the sound pressure level distribution. In the ZX plane, the maximum sound pressure level is distributed in the middle section of the ZX with a value of 98.6 dB, and the minimum sound pressure level also appears in the middle section of the ZX plane with a value of 65 dB. The acceleration distribution near the two sides of the shell is relatively dense, indicating that there is more superposition of sound waves in this region, which has a greater influence on the sound pressure level distribution.

Combined with the overall surface maximum deformation and surface acceleration, it can be seen that under the excitation of the incident wave, the acoustic waves are conducted on the surface of the shell and will be repeatedly superimposed in some parts, thus generating more energy and a larger sound pressure level is generated. The maximum deformation occurs at the site where the maximum sound pressure level exists in all three planes, and the acceleration distribution on the surface is also denser, because the magnitude of the surface acceleration can influence the acoustic wave transmission, which can lead to micro-deformation of the surface. To reduce the surface acceleration, acoustic cotton can be added to the inner cavity of the shell or further structural optimization can be carried out.

### Sound pressure level distribution and structure deformation under different incident frequencies

**Figure 3:**
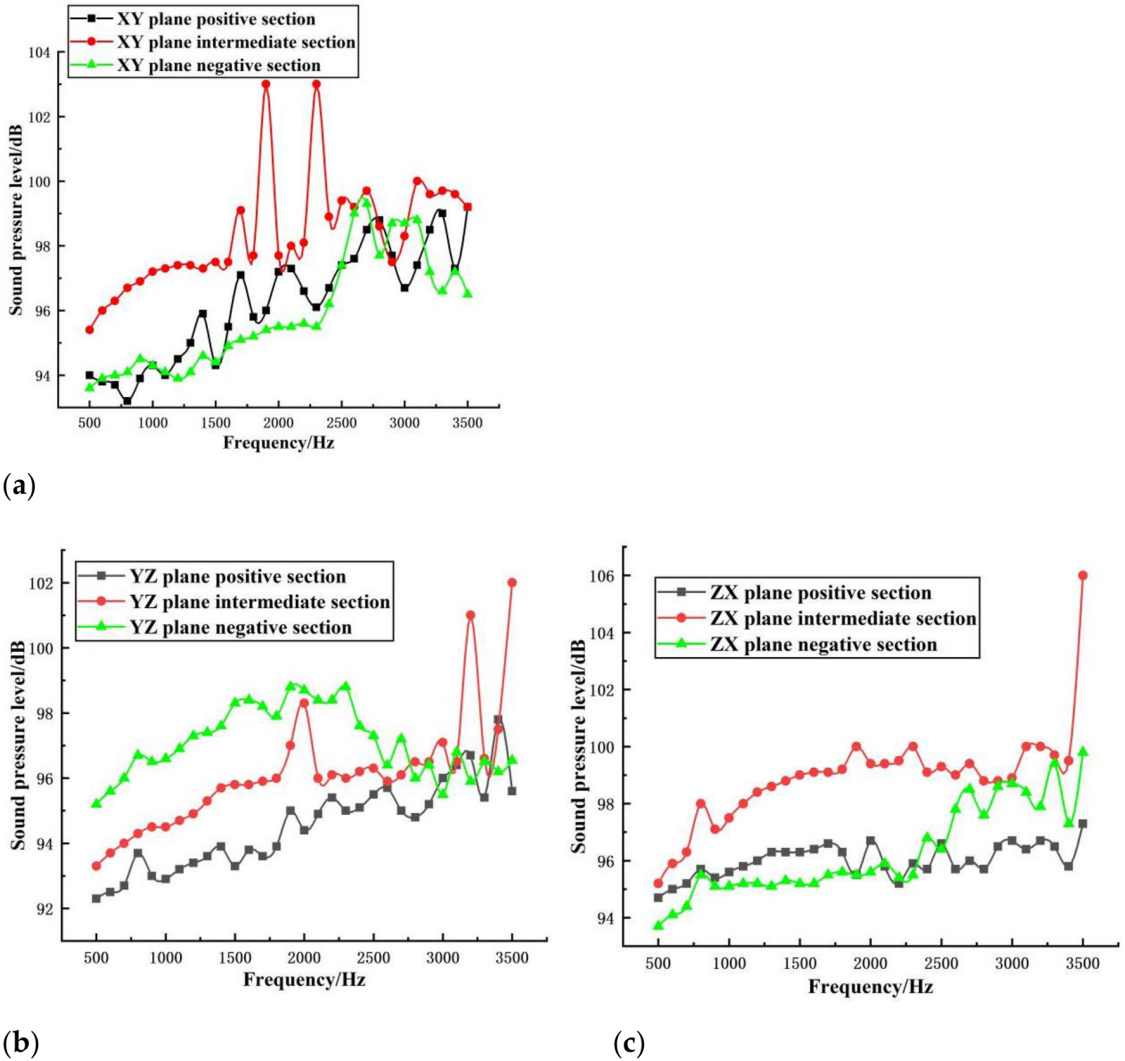
Sound pressure level distribution at different incident frequencies. (**a**) Sound pressure level distribution in each plane of XY plane, (**b**) Sound pressure level distribution in each plane of YZ plane and (**c**) Sound pressure level distribution in each plane of ZX plane.

**Figure 4:**
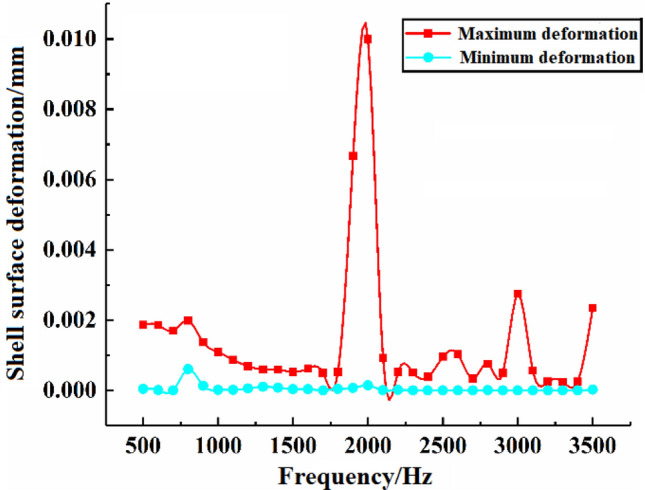
Structural deformation at different incident frequencies.

After calculation, the first six structural modes of the compressor shell are 716.3 Hz, 1102.8 Hz, 1514.4 Hz, 1994.1 Hz, 2581.5 Hz and 2857.3 Hz, and the first six acoustic modes are 509.79 Hz, 648.32 Hz, 681.93 Hz, 908.27 Hz, 928.7 Hz and 1033 Hz, respectively. In order to analyze some basic laws of acoustic-structural coupling more comprehensively, the external acoustic pressure excitation frequency is extended to 500~3500 Hz. meanwhile, the sound pressure levels and the structural deformation laws of incident waves of different frequencies excited in each plane were analyzed, and the results are shown in Figs. 3 and 4.

The distribution of the sound pressure levels in the three directions and the variation of the sound pressure levels in different frequency bands are shown in Fig. 3.

On the whole, the maximum sound pressure level appears in each plane to different degrees, and the maximum sound pressure level in each plane corresponds to different incident frequencies. However, the maximum sound pressure level generated by individual incident waves appears in multiple planes at the same time, because the location where the maximum sound pressure level can be generated does not completely transmit the sound, and the sound is constantly superimposed and reflected in that region.

Specifically, in the XY plane, the sound pressure level generated in the middle plane is greater than that in the other two planes in the 500–1500 Hz incoming frequency band, and the sound pressure levels in the other two planes vary alternately. In the 1500–2600 Hz incoming frequency band, the highest sound pressure level appears in the middle plane, with a value of 103 dB, while the sound pressure level in the positive plane is the next highest, and the negative plane is the smallest. This is because the middle plane is the densest area of acoustic superposition when producing acoustic reflection. In the YZ plane, in the 500–2600 Hz incoming frequency band, the overall sound pressure level generated by the negative section is greater than that of the other two sections, and the sound pressure level of the positive section is the smallest. The highest sound pressure level appears in the high frequency region of 2600–3500 Hz, and its value is 101 dB. In the ZX plane, in the whole frequency band, the sound pressure level of the middle section is greater than that of the other two sections, and the highest sound pressure level appears in the middle section at 3500 Hz with a value of 106 dB, while in the 2100–2400 Hz band, the sound pressure level of the negative section alternates with that of the positive section, and the value of the negative section is greater than that of the positive section in other frequency bands.

At the specific incident wave pressure, the deformation law of the shell structure at different incident frequencies is shown in Fig. 4. In the shell surface, each frequency corresponds to the maximum and minimum deformation. From the whole frequency band, the fluctuation of the maximum deformation of the shell surface is large, and at 1900 HZ, the maximum deformation is produced with the value of 0.007 mm, while the fluctuation of the minimum deformation of the shell surface is small, and its value is basically around 0.0008 mm.

### Sound pressure level and structure deformation law under different incident wave pressure

**Figure 5:**
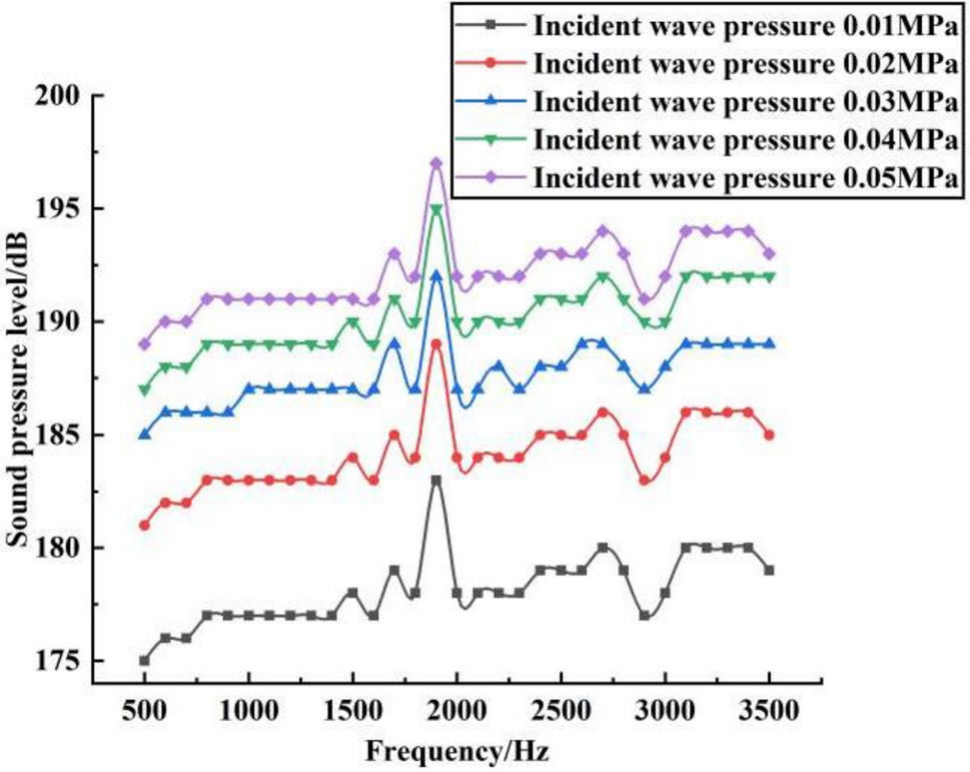
Sound pressure levels under different incident wave pressures.

**Figure 6:**
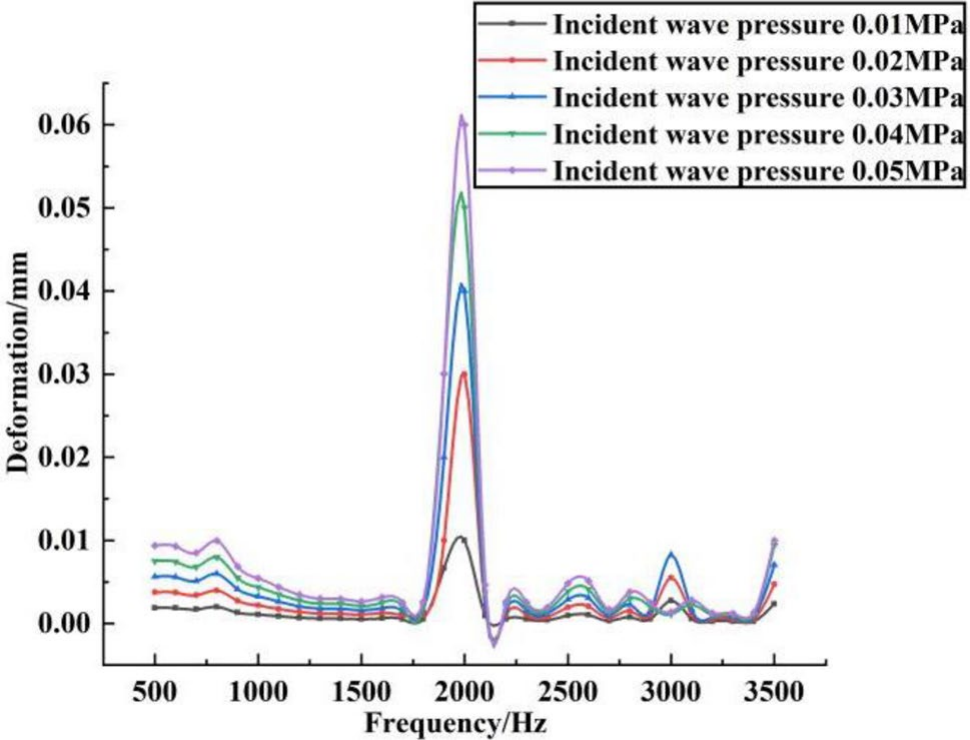
Shell deformation under different incident wave pressures.

Based on the analysis of the effects of different incident frequencies on the sound pressure levels in different planes of the shell, the effects of different incident pressures on the shell sound pressure levels and the shell deformation were analyzed, as shown in Figs. 5 and 6, respectively.

From Fig. 5, the maximum sound pressure level on the shell tends to increase slightly in the frequency band 500–3500 Hz under the same incident wave pressure, but the change fluctuation is relatively small. And at the same frequency, with the increase of incident wave pressure, the maximum sound pressure level on the shell also increases, which reaches the maximum sound pressure level near 2000 Hz. And in the frequency range of 500–3500 Hz, the trend of sound pressure level changes in different incident wave pressure is basically the same. This is because the magnitude of the incident wave pressure affects the magnitude of the acceleration on the surface of the shell, which affects the change in the sound pressure level. Secondly, as the incident wave pressure increases, the superposition of its acoustic waves inside the shell is produced more strongly, which leads to an increase in the sound pressure level. As can be seen from Fig. 6, the effect of frequency variation on shell deformation is not obvious in the frequency band 500–3500 Hz. And at the same frequency, the deformation on the shell increases with the increase of the incident pressure wave. The shell deformation patterns at different incident wave pressures are basically the same, where the deformation at the frequency of 2000 Hz is the largest at the same incident wave pressure. This is because the size of the incident wave pressure will have some effect on the shell structure, especially in the case of relatively strong acoustic resonance size near 2000 Hz, which is more likely to cause relatively large deformation.

## Experimental research and analysis

**Figure 7:**
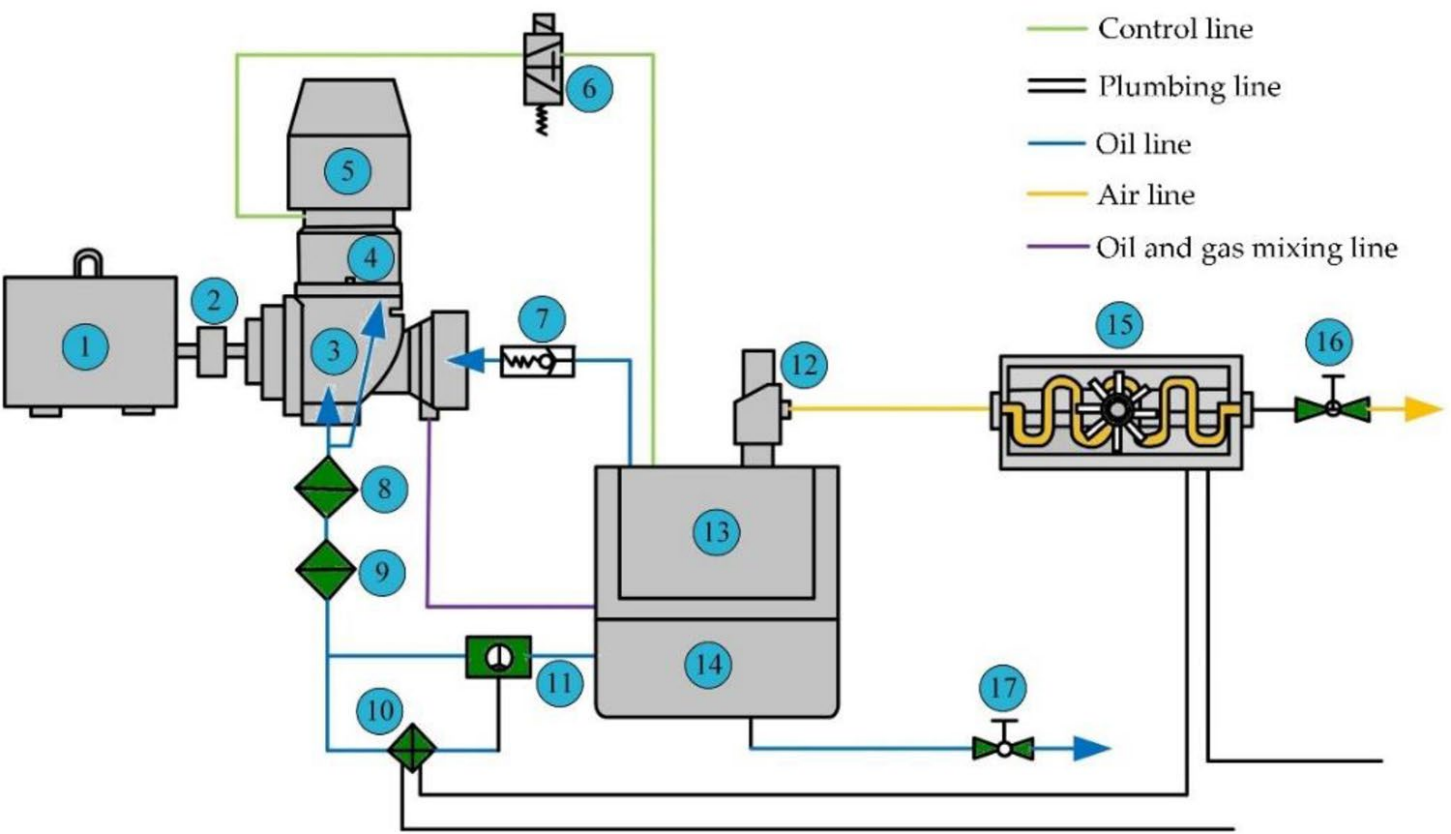
Test platform system diagram.

In order to verify the correctness of the established twin-screw air compressor model and simulation method, a twin-screw air compressor with the same technical parameters as the model constructed in the paper was used as the test object. The whole test system consists of the motor, the main engine of the screw air compressor, the oil–gas separator and the cooler and other supporting equipment. The test platform system is shown in Fig. 7.

Serial number in the figure: 1 Electric motor 2 Coupling 3 Mainframe 4 Intake valve 5 Air filter 6 Solenoid valve 7 One-way valve 8 Oil shut-off valve 9 Oil filter 10 Oil cooler 11 Temperature control valve 12 Minimum pressure valve 13 Precision oil and gas separation core 14 Oil and gas drum 15 Aftercooler 16 Ball valve 17 Oil drain valve.

### Working principle of the test system

No-load test before the test, that is, running at rated speed for 10 min, after passing the no-load test before the test. After the motor is started, the main compressor starts to run, and the clean air is filtered by the air filter into the main compressor. The main machine compresses continuously and outputs a high temperature, high pressure oil and gas mixture, which flows into the oil and gas drum through the oil pipe. Due to the difference of oil and gas density, it will be separated automatically in the oil and gas barrel. There are two paths after separation: one is the oil pipeline, the oil sunk into the bottom of the oil and gas drum flows into the oil cooler through the temperature control valve, after cooling the lower temperature oil is filtered through the oil filter back to the compressor to continue recycling; the other is the air pipeline, the oil and gas mixture after entering the oil and gas drum, through the oil and gas separator to further separate the oil and pure air. The separated pure air passes through the minimum pressure valve and part of it flows into the compressor for further circulation, while the excess gas is discharged through the aftercooler for cooling. The separated oil, through the check valve in the oil pipeline, enters the compression mainframe for reuse.

**Figure 8:**
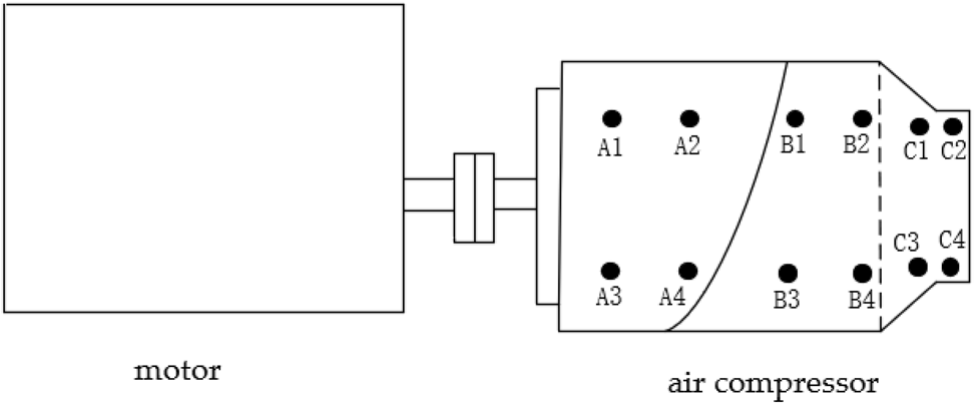
Layout of measurement point locations.

**Figure 9:**
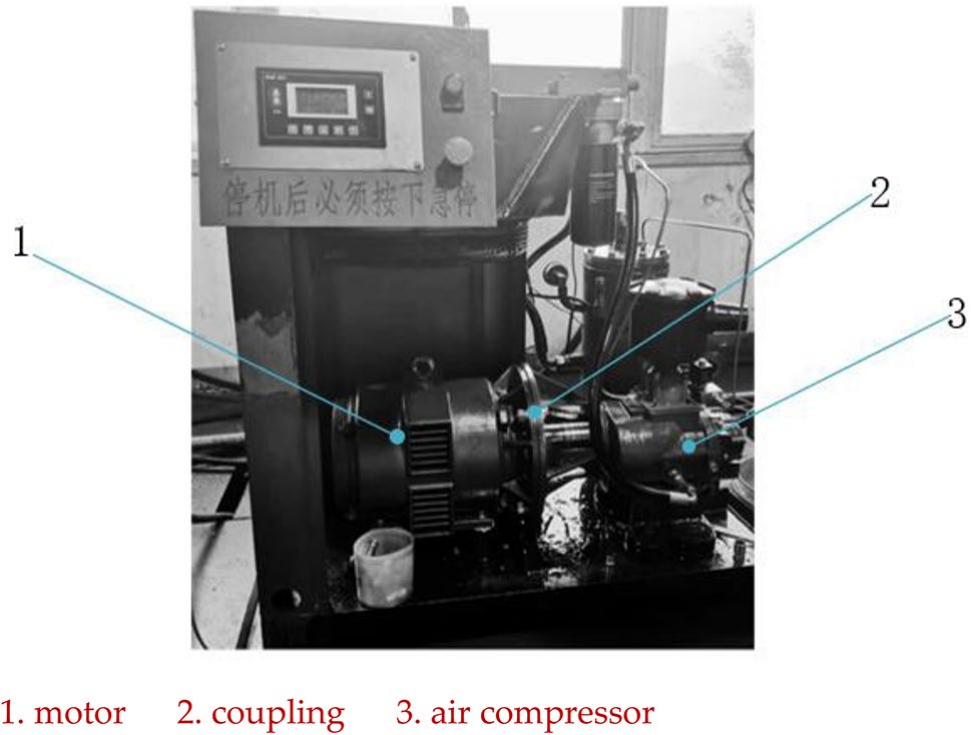
The test system.

In the experiment, different frequencies of the compressor system were excited by setting different rotational speeds. In order to obtain the data required for the test, the displacement values at different locations of the screw compressor mainframe were collected using a vibration measuring instrument, and the average value of deformation experimental data was obtained. In the experiment, in order to obtain the displacement data of the compressor shell as comprehensively as possible, according to the shape characteristics of the shell, it is divided into three parts, such as A, B and C. At the same time, four measurement points are arranged in each part, and the specific location of the measurement points is shown in Fig. 8. An all-in-one vibration measuring instrument was applied for the measurement, which was modeled as AR63A with a displacement measurement range of 0.001–1.999 mm. During the measurement, the probe of the instrument was moved to the position of the measurement point and the data displayed by the instrument was recorded. Figure 9 shows the site diagram of the test system.

### Comparison analysis of experimental data and simulation results

In order to verify the correctness of the simulation, the shell deformation at the incident wave pressure of 0.02 Mpa was experimentally tested. When the incident wave pressure is 0.02 Mpa, the average value of the shell deformation result obtained from simulation is 0.021 mm, while the shell deformation measured from test is 0.020 mm. It can be seen that the deformation values of the shell obtained from the simulation are larger than the actual measured values, which is due to the fact that the muffler was installed in the test system, but not added in the simulation. Considering the performance of the muffler in the experimental system, the simulation under the same working conditions after adding the muffler is done. The muffler is an impedance composite muffler with a minimum transmission loss value of 21.5 dB and a maximum transmission loss value of 29.2 dB. The average value of the deformation of the shell obtained from the simulation is 0.019 mm, which is smaller than the actual measured value of 0.020 mm, and at this time, the difference between the simulation and the experimental results is 5.0%. This is due to the fact that in the experimental system, there is micro-deformation of the air compressor shell caused by rotor grinding due to mounting errors, wear, etc.

## Conclusion

In this paper, a multi-physics field coupling simulation technique is used to simulate the sound pressure level and structural deformation law on the surface of twin-screw air compressor head shell with external excitation, and the specific conclusions are as follows.

1. Under the same incident wave pressure, the distribution pattern of sound pressure level in different planes of the whole shell is different, and the distribution of sound pressure level in different sections of the same plane is also different. Among them, the maximum sound pressure level distribution on the whole shell surface is in the middle section of ZX with the value of 98.6 dB, and the minimum sound pressure level appears in the middle section of YZ plane with the value of 56.5 dB.
2. From the distribution of sound pressure level at different frequencies of the same incident wave, it can be seen that in the XY plane, in the 1500–2600 Hz incident frequency band, the highest sound pressure level appears in the middle plane, and its value is 103 dB; in the YZ plane, the maximum sound pressure level appears in the range of 2600–3500 Hz, and its value is 101 dB; In the ZX plane, the highest sound pressure level appears in the middle section with the frequency of 3500 Hz, and the value is 106 dB. While the shell deformation pattern is not obvious under this condition, and it is basically in a small fluctuation state.
3. Under different incident wave pressure, when the incident wave pressure increases, the sound pressure level of the whole shell surface also increases, and when the incident wave pressure is 0.05 MPa, the maximum sound pressure level appears near 1900 Hz, and its value is 197 dB; at the frequency of 2000 Hz, the deformation of the shell is the largest at the same incident wave pressure, and its value is 0.06 mm.

The results of this paper have some reference significance for the optimal design of air compressor casing structure to improve the operating efficiency and reduce energy consumption of the air compressors.

## Data Availability

All data generated and analyzed during this study are included in this published article.
